# Nocturnal haemoglobin oxygen saturation variability is associated with vitamin C deficiency in Tanzanian children with sickle cell anaemia

**DOI:** 10.1111/j.1651-2227.2010.02078.x

**Published:** 2011-04

**Authors:** SE Cox, V L'Esperance, J Makani, D Soka, CM Hill, FJ Kirkham

**Affiliations:** 1 London School of Hygiene & Tropical MedicineLondon, UK; 2 Muhimbili University of Health and Allied SciencesDar-es-Salaam, Tanzania; 3 Division of Clinical Neurosciences, School of Medicine, University of SouthamptonUK; 4 University College London Institute of Child HealthUK

**Keywords:** Obstructive sleep apnoea, Oxidative stress, Oxygen saturation, Sickle cell anaemia, Vitamin C deficiency

## Abstract

**Aim:**

To compare pulse oximetry in children with sickle cell anaemia (SCA) and controls and test the hypothesis that vitamin C deficiency (VCD; <11.4 μmol/L) is associated with nocturnal haemoglobin oxygen desaturation in SCA.

**Methods:**

We undertook nocturnal and daytime pulse oximetry in 23 children with SCA (median age 8 years) with known steady-state plasma vitamin C concentrations and 18 siblings (median 7 years).

**Results:**

Median nocturnal delta 12 s index (delta12 s), a measure of haemoglobin oxygen saturation (SpO_2_) variability, was 0.38 (interquartile range 0.28–0.51) in SCA and 0.35 (0.23–0.48) in controls, with 9/23 and 6/18, respectively, having a delta12 s >0.4, compatible with obstructive sleep apnoea (OSA). Eleven of twenty-three with SCA had VCD; logged vitamin C concentrations showed a 66% decrease per 0.1 unit increase in delta12 s ([95% CI −86%, −15%]; p = 0.023) and delta12 s >0.4 was associated with VCD (odds ratio 8.75 [1.24–61.7], p = 0.029). Daytime and mean nocturnal SpO_2_ were lower in SCA but there was no association with vitamin C.

**Conclusion:**

Obstructive sleep apnoea (OSA), detected from nocturnal haemoglobin oxygen saturation variability, is common in Tanzanian children and associated with vitamin C Deficiency in SCA. The direction of causality could be determined by comparing OSA treatment with vitamin C supplementation.

## Introduction

Erythrocytes containing haemoglobin S (HbS) experience chronic redox imbalance from increased production of hemichromes and therefore reactive oxygen species (ROS). The associated haemolysis contributes to many of the pathophysiological pathways in sickle cell anaemia (SCA), potentially mediated by oxidant stress, decreased nitric oxide bioavailability, inflammation and hypoxia. The compromise of endothelial function may be exacerbated by intermittent nocturnal hypoxia (1) associated with obstructive sleep apnoea (OSA), common in SCA (2). The delta 12 s index (delta12 s), the absolute difference in haemoglobin oxygen saturation (SpO_2_) between successive 12-s intervals, measures baseline SpO_2_ variability. In adults in the general population, delta12 s values of >0.4 predict an apnoea/hypopnea index (AHI) of >15 with 88% specificity and 70% sensitivity (3). In 71 children with sickle cell disease enrolled in the Sleep Asthma cohort (4), using the same cut-offs for delta12 s and AHI, specificity and sensitivity were 100% and 89%, respectively (Gavlak et al., unpublished).

OSA is associated with oxidative stress and endothelial dysfunction (5). Supplemental vitamin C improves endothelial function in OSA in adults in the general population (6). Although vitamin C deficiency (VCD) appears to be common in SCA children (7), the possibility of an association with low SpO_2_, either intermittent or chronic, has not been explored. To test the hypothesis that low antioxidant status is associated with intermittent and/or chronic hypoxia in children with SCA, we undertook overnight pulse oximetry in well SCA children who were enrolled in an African urban cohort and sibling controls; those with SCA also had steady-state vitamin C levels measured.

## Methods

Ethical permission was granted by the Muhimibili University of Health & Allied Sciences ethics committee (Ref: MU/RP/AECNoI.XII/77). Children were recruited from confirmed HbSS patients enrolled in a cohort study at Muhimbili National Hospital, Dar-es-Salaam, from April to July 2009 and their siblings. They were not selected as having sleep or breathing problems. Informed consent was obtained from parents of the children; where appropriate, assent was obtained from children themselves. Pulse oximetry was sampled in the day at rest and over a single night using a 2-s averaging time and 1 Hz sampling rate (Masimo Radical, Irvine, CA, USA). Data analysis was performed with Download 2001 software (Stowood Scientific, Oxford, UK). Poor perfusion, low signal IQ and movement artefact data were rejected. Analysis software yielded standard measures including mean and minimum SpO_2_, delta12 s and desaturation index of 3% or greater from baseline. Analyses of artefact-free recordings were conducted and data were compared between children with SCA and their siblings using the independent *t*-test for normally distributed data or the non-parametric Mann–Whitney *U*-test. Steady-state vitamin C concentrations were measured using a fluorometric method by Human Nutrition Research, Cambridge, UK, in plasma samples separated and stabilized within 2 h of collection with metaphosphoric acid.

In the children with SCA, associations between logarithmically transformed vitamin C concentrations and oximetry variables were assessed using linear regression and by logistic regression of VCD and binary oximetry data. All oximetry variables were tested for associations with the potential covariates: age, sex, body mass index (BMI)-*z*-score for age and averaged steady-state haemoglobin, from data collected at routine clinic visits and entered into the cohort study database.

## Results

Eighteen control siblings, six boys, median age 7 (range 2–12) years, underwent overnight pulse oximetry, as did 23 children with SCA, 13 boys, median age 7.8 (range 2.9–15.1) years, who had had steady-state vitamin C concentrations measured prior to the sleep study. Ethics was not granted for venepuncture in the controls.

Descriptive statistics for haemoglobin and pulse oximetry data in controls and children with SCA and for steady-state vitamin C in those with SCA are given in Table 1. Daytime haemoglobin oxygen saturation was lower in the children with SCA than in the controls and there was a trend for lower mean nocturnal haemoglobin oxygen saturation but there was no difference between children with SCA and controls in sleep duration, minimum overnight SpO2, number of overnight SpO_2_ dips >3%/hr and the delta 12 s index (Table 1).

**Table 1:** Vitamin C, steady-state haemoglobin and pulse oximetry data in 23 children with SCA and 18 control siblings

Variable	Children with SCA	Control siblings	p
Median steady-state plasma vitamin C μmol/L	12.5 (IQ range 3.1–24.1)		
Mean steady-state haemoglobin* (g/dL)	7.7 (SD 1.16) (range 5.8–10.9)		
Mean daytime SpO_2_ (%)	96.0 (SD 3.68) (range 85–100)	98.6 (SD 1.95) (range 93–100)	0.005
Median recorded study duration (h)	9.47 (IQ range 7.78–10.82)	9.37 (IQ range 5.98–11.06)	0.8
Mean overnight SpO_2_ (%)	96.3 (SD 4.06) (range 77.9–100)	97.9 (SD 2.0) (range 91.4–99.9)	0.1
Mean minimum overnight SpO_2_ (%)	85.3 (SD 9.3) (range 65–95)	85.9 (SD 9.1) (range 63–95)	0.8
Median delta 12 s Index	0.38 (IQ range 0.28–0.51)	0.35 (IQ range 0.23–0.48)	0.25
Median number of overnight SpO_2_ dips > 3%/h	5.19 (IQ range 1.09–7.64)	2.44 (IQ range 1.11–6.16)	0.4

* Mean of multiple measurements made at between 3 and 15 routine steady-state (no fever, reported pain, malaria parasites or antigens, or admission within 90-day period) routine clinic visits preceding sleep study.

SpO_2_ = haemoglobin oxygen saturation; IQ range = interquartile range.

Forty-eight per cent (11/23) of children with SCA had VCD (<11.4 μmol/L), a similar proportion to all patients with SCA with data available (58%; 463/799) but higher than the proportion in a historical group of Tanzanian control children (32%, 24/74) (Cox et al., unpublished data). There was no association between vitamin C and age, sex, nutritional status (BMI-*z*-score) or steady-state haemoglobin in the children with SCA.

In the children with SCA, geometric mean vitamin C decreased by 66% per 0.1 unit increase in delta12 s ([95% CI −86%/−15%] p = 0.023) but delta12 s was not associated with duration of sleep, age, sex, BMI-*z*-score or steady-state haemoglobin. Vitamin C concentration also decreased with higher numbers of episodes of SpO_2_ desaturations >3%/h (6.2% decrease [95% CI −11.8%/−2.5%], p = 0.042). There were no associations with vitamin C and the other oximetry variables. A high delta12 s (>0.4) was significantly associated with an odds ratio for VCD of nearly nine times greater (Table 2).

**Table 2:** Logistic regression between abnormal oximetry measures and steady-state vitamin C deficiency, N=23

Variable	Vitamin C sufficient (%)	Vitamin C deficient (%)	OR [95% CI], p value
Mean daytime SpO_2_ <96%	3/12 (25)	6/11 (54.6)	3.60 [0.62 – 21.03], p = 0.155
Mean overnight SpO_2_ <94%	2/12 (16.7)	2/11 (18.2)	1.11 [0.13 – 9.61], p = 0.92
Delta 12 s Index >0.4	2/12 (16.7)	7/11 (63.7)	8.75 [1.24 – 61.7], p = 0.029
Overnight SpO_2_ dips (> 3%) >4/h	5/12 (41.7)	8/11 (72.7)	3.77 [0.65 – 21.6], p = 0.14

SpO_2_ haemoglobin oxygen saturation

## Discussion

In line with previous data (1), our study reports lower daytime and mean nocturnal haemoglobin oxygen saturation in children with SCA than in controls, although the latter did not reach statistical significance. OSA is commoner in black children, but the limits acceptable as within the normal range have not been defined in this population (8). There are few data comparing measures of desaturation and OSA between children with SCA and ethnically matched controls, although in one study, mean and minimum overnight SpO_2_ of <95.8 and <80%, respectively, were not seen in 50 controls, half of whom were siblings (2). Interestingly, in our data, 2 and 3 controls, respectively, had values below the mean and minimum overnight SpO_2_ in Samuels’ study of children living in England (2). In addition, there was no difference between children with SCA and sibling controls in study duration, minimum overnight SpO_2_, the number of SpO_2_ dips >3% and delta12 s, a measure of the variability of SpO_2_ predictive of OSA in adults in the general population and children with SCA. Our sample was small, and further studies should explore genetic and environmental factors, including VCD, in children with SCA and ethnically matched controls, both siblings and unrelated.

This is the first report to test for an association between vitamin C status and nocturnal oximetry measures in SCA. We hypothesized that intermittent and/or chronic nocturnal hypoxia would be associated with low vitamin C status, because of the effects of associated oxidant stress. Delta 12 s correlated with vitamin C concentrations in children with SCA and VCD was associated with greatly increased odds of a high delta12 s. OSA is known to cause intermittent hypoxia, and vitamin C concentrations were also inversely correlated with the number of dips in SpO_2_ >3%/h, although with VCD and the number of dips in SpO_2_ >3%/h dichotomized this relationship did not reach statistical significance.

There is evidence for links between VCD and endothelial function in adults with OSA and in children with SCA. In a study by Grebe et al. (6), patients with untreated OSA had significantly reduced endothelial-dependent vasodilation compared to controls, an effect suggested to be mediated via increased oxidant stress. Intravenous vitamin C supplementation increased endothelial vasodilation in the patients with OSA but had no effect in controls; unfortunately, vitamin C concentrations were not reported in that manuscript. In another study of adult patients with SCA, not investigated for OSA, vitamin C oral supplementation (300 mg/day) for 6 weeks decreased forearm vascular resistance as well as increasing forearm blood flow and the vasodilator effect of warmth stimulation (9). Patients with SCA may have increased antioxidant requirements because of production of ROS from unstable erythrocytes. Exposure of sickle erythrocytes to vitamins C & E *in vitro* reduced markers of erythrocyte oxidant stress (10). In addition, markers of lung function have been associated with the percentage of irreversibly sickled cells (11), which was shown by the same group to be decreased by vitamin C supplementation (12).

Our study suggests a link between SpO_2_ variability and vitamin C deficiency in SCA. However, it is not possible to conclude that low vitamin C concentrations are a result of SpO_2_ variability and hypoxia and not causal. Vitamin C may be important in the response to hypoxia, through its role in carotid body sensitivity (13) and stabilization by vitamin C-dependent prolyl-hydroxylase enzymes of hypoxia inducible factor 1 (HIF-1), which regulates acute and chronic hypoxic responses (14). In support of low vitamin C being causal for intermittent hypoxia is the observation that vitamin C supplementation reversed age-associated depression in the hypoxic hyperventilatory response in elderly subjects (15). Differences in the hypoxic hyperventilatory response might be important in adaptations to both chronic and intermittent hypoxia in conditions, such as SCA, in which OSA is also a feature. Further studies adequately powered to examine measurements of SDB other than the delta 12 s index in children with SCA, and including vitamin C measurement in control children with and without OSA, are justified. To guide future therapeutic interventions, the direction of causality could be tested by investigating the effect of continuous positive airway pressure treatment on vitamin C concentrations (16) and the effect of vitamin C supplementation on OSA and responses to hypoxia.

## Abbreviations

AHI: Apnoea/hypopnea index
BMI: Body mass index
CPAP: Continuous positive air pressure
delta12 s: Delta 12 second index
FEV1: Forced expiratory volume at 1 second
Hb: Haemoglobin
MCHC: Mean cell haemoglobin concentration
OSA: Obstructive sleep apnoea
ROS: Reactive oxygen species
SCA: Sickle cell anaemia
SpO2: Haemoglobin oxygen saturation
VCD: vitamin C deficiency
